# High Household Transmission Among Asymptomatic Contacts Across Pandemic Waves in Cincinnati, Ohio

**DOI:** 10.3390/epidemiologia6040091

**Published:** 2025-12-12

**Authors:** Katherine Bowers, Stefanie Benoit, James Rose, Andrew F. Beck, Alonzo T. Folger, Tara N. Calhoun, Melissa E. Day, Andrew Lovell, Maryse Amin

**Affiliations:** 1 Division of Biostatistics and Epidemiology, Cincinnati Children’s Hospital Medical Center, Cincinnati, OH 45229, USA; 2 Department of Pediatrics, College of Medicine, University of Cincinnati, Cincinnati, OH 45267, USAtara.calhoun@cchmc.org (T.N.C.);; 3 Division of Bone Marrow Transplantation & Immune Deficiency, Cincinnati Children’s Hospital Medical Center, Cincinnati, OH 45229, USA; 4 Division of Nephrology and Hypertension, Cincinnati Children’s Hospital Medical Center, Cincinnati, OH 45229, USA; 5 Division of General and Community Pediatrics, Cincinnati Children’s Hospital Medical Center, Cincinnati, OH 45229, USA; 6 Division of Hospital Medicine, Cincinnati Children’s Hospital Medical Center, Cincinnati, OH 45229, USA; 7 Division of Infectious Diseases, Cincinnati Children’s Hospital Medical Center, Cincinnati, OH 45229, USA; 8 Cincinnati Health Department, Cincinnati, OH 45229, USA

**Keywords:** COVID-19, SARS-CoV-2, household contact, serology

## Abstract

Background/Objectives: COVID-19 and long COVID remain prevalent, with household transmission being an important mode of spread. To quantify household transmission of subclinical SARS-COV-2 infection and identify sociodemographic risk factors that may explain disparities in transmission, we conducted a case-ascertained antibody surveillance study of households in Cincinnati, Ohio. Methods: A partnership was formed between the Cincinnati Health Department and Cincinnati Children’s Hospital Medical Center. The Health Department identified cases of COVID-19. Infected individuals, along with their household contacts (n = 245), completed multiple questionnaires about symptoms, demographics, psychosocial (Adverse Childhood Experiences Scale and Everyday Discrimination Scale) and social risk factors, and conditions before and during the pandemic. In addition, they completed a non-fasting blood draw for IgG, IgM, IgA, and nucleocapsid protein serology testing. Results: Household contacts experienced few symptoms of COVID-19. However, according to the presence of the nucleocapsid protein, nearly 50% contracted the SARS-CoV-2 virus. This rate was similar by vaccination status but it was higher for household contacts who experienced high levels of early life adversity compared with those with lower levels. Conclusions: Our results confirm the high transmission of subclinical disease among household contacts, which may vary due to psychosocial factors. This reinforces the importance of isolating cases to prevent transmission, regardless of vaccination status.

## 1. Introduction

In December 2019, an outbreak of coronavirus disease (COVID-19), caused by the Severe Acute Respiratory Syndrome Coronavirus-2 (SARS-CoV-2), emerged as a global pandemic [1]. The first cases in the Greater Cincinnati region were identified by March of 2020. Even now, several years later, cases of acute disease as well as cases of long COVID continue to affect children and adults [2]. Reported cases of long COVID remain prevalent [3], and a comprehensive understanding of the household transmission of SARS-CoV-2, leading to recommendations for prevention, remains an important area of research.

Transmission characteristics vary by strain and are also likely to vary by household- and other individual- and community-level characteristics [4]. In addition, COVID-19 continues to disproportionately affect racial and ethnic minorities. A 2021 systematic review and meta-analysis found that COVID-19 positivity is highest in those identifying as Black/African American and those identifying as Hispanic, compared with those identifying as White, American Indian or Alaska Native, Asian American, Pacific Islander, and multiracial or another race/ethnicity.

Presentation and severity characteristics have also been shown to vary across sociodemographic factors. Intensive care unit admission was highest for Asian Americans [5]. Additionally, living in a deprived neighborhood (measured using a deprivation index) was associated with mortality in Asian American and Hispanic individuals. Decreased access to medical care was associated with COVID-19 positivity rates for both Hispanic and African American individuals [5]. Similar differences in disease severity have been observed in Cincinnati. From 2020 to 2024, the Black incidence rate was 5980.9 per 100,000 residents, while the rate was 4930.7 per 100,000 for White residents [6]. In addition, between 2020 and 2024, the Black hospitalization rate was 338.7 per 100,000 compared to 166.6 for White—a disparity ratio of 2.03.

However, less is known about how additional psychosocial factors, such as early life adversity and experiences with discrimination, may relate to COVID-19 transmission.

The objective of our study was to quantify the percentage of subclinical cases of COVID-19 among household contacts in Cincinnati, Ohio, and to determine whether rates of subclinical infection varied by race, ethnicity, as well as by sociodemographic and psychosocial characteristics. Since the vaccine was developed and widely distributed during the course of our study, we additionally employed serology of the nucleocapsid protein to distinguish between vaccine and viral response [7]. With the understanding that race is a sociopolitical construct, indicative of the experience of racism in our structures and systems, we hypothesized that self-reported race would be associated with higher transmission of SARS-CoV-2.

## 2. Materials and Methods

We conducted a case-ascertained antibody-surveillance study of households in Cincinnati, Ohio. To understand the transmission dynamics in Cincinnati, Ohio, a partnership was formed between Cincinnati Children’s Hospital Medical Center (CCHMC) and the Cincinnati Health Department (CHD). We enrolled index cases and their household contacts for participation in the study. Beginning monthly in November of 2021, the CHD compiled a list of reported infections. Since COVID-19 cases were reportable events, the CHD had a comprehensive list of test-positive SARS-CoV-2 infections in the city.

We employed stratified randomization to the lists of reported infections to determine the order of recruitment. Randomization was stratified by race and ethnicity to increase the likelihood of enrolling non-Hispanic Black, non-Hispanic White, and Hispanic participants, the predominant racial and ethnic groups in the region. The research assistant at CHD would contact one potential participant from each race and ethnicity (non-Hispanic Black, non-Hispanic White, Hispanic) and then move on to the next racial category for recruitment. The first contact was with index cases unless the index case was a child, in which case a parent was contacted. At the time of study initiation, CHD was involved with all contact tracing. CHD partners noted that contact tracing was able to contact 85% of individuals with whom tracing was attempted. From the list of close contacts, we assumed they were household contacts if a close contact had an identical address. The research assistant would call up to three times, leaving a voicemail followed by a single text message if the contact was unable to be reached. If a case agreed to participate in the study, their contact information was shared with research coordinators at CCHMC. All adult participants provided written informed consent utilizing an electronic consent (e-consent). Children ages 11–15 years provided verbal assent, and children 16–17 years provided written assent.

Inclusion criteria included index cases with past COVID-19 infection or household contact of an individual with a past infection. The date of infection must have occurred between one and three months prior to enrollment. An index case included the first positive in a household with a PCR- or antigen-test confirmed infection or a probable case with two or more symptoms and an epidemiologic link (at the time the study was initiated, probable cases were 3% of index cases). Index cases must have had household contacts and resided within the city of Cincinnati, Ohio, to be eligible. Household contacts included relatives or non-relatives living in the same house or apartment. Exclusion criteria included individuals living in congregate care facilities (e.g., nursing homes, group homes, shelters for the unhoused, prisons).

### 2.1. Study Visit

After providing written e-consent, participants completed several assessments electronically using REDCap’s Survey function. The assessments included demographics, early life adversity, everyday discrimination (ages 13 and older with parental permission for minors), medical history, COVID-19 symptoms, and COVID-19 vaccination history (i.e., whether they were offered and/or had any COVID-19 vaccines). Early life adversity was measured using the adverse childhood experiences (ACE) scale (adult participants), a 10-item scale that captures various types of trauma experienced during childhood [8]. Discrimination was captured using the Everyday Discrimination Scale which measures the frequency of day-to-day experiences of discrimination using a scale ranging from ‘almost every day’ to ‘never’ [9]. For those answering ‘a few times a year’ or more frequently, they were then asked the main reason for the discrimination (race, age, gender, etc.). The internal consistency of both scales was high, with Cronbach’s alpha of 0.88 for the ACE scale [8] and 0.88 [9] for the Everyday Discrimination Scale, based on prior validation studies. To better understand how each participant was affected by the pandemic, questions were asked about their experience before and during the pandemic. For example, participants were asked about food insecurity in the 12 months before the pandemic and their current experiences. When the study was initiated, the vaccine was not developed, and the study was modified once vaccines were available. In addition to remote assessments, study participation included a one-hour-long study visit to the Schubert Research Clinic at CCHMC with a non-fasted blood draw. If assessments were not completed remotely, they were administered at the study visit via iPad. In the event of technical difficulties, the assessments were administered via pen and paper. Participants were provided a monetary incentive for their time.

### 2.2. Laboratory Analyses

To evaluate the immunologic characteristics of index cases and to determine whether household contacts contracted the SARS-CoV-2 virus, serologic testing was conducted. This also enabled us to evaluate the antibody response to the virus. Specifically, to identify subclinical infection, we determined the presence of IgG, IgM, IgA, and the nucleocapsid protein [10]. One 5 mL sample of whole blood was collected at the Schubert Research Clinic and then transferred to the CCHMC Nephrology Clinical Laboratory for processing and analyses. Samples were centrifuged, and plasma and/or serum were extracted and stored at −80 degrees Celsius until serologic assays were performed. Analyses included serology tests for SARS-CoV-2 IgG, IgM, and IgA, and nucleocapsid testing. IgG was measured via enzyme-linked immunosorbent assay [EUROIMMUN, Mountain Lakes, NJ, USA; (Cincinnati Children’s Hospital Medical Center, Cincinnati, OH, USA, USA, Lab Developed Test)] or chemiluminescent immunoassay (DIASORIN, Stillwater, MN, 311460), depending upon the time of specimen testing. IgM was measured via enzyme-linked immunosorbent assay (Cincinnati Children’s Hospital Medical Center, Cincinnati, OH, USA, Lab Developed Test) or chemiluminescent immunoassay (DIASORIN, Stillwater, MN, USA, 311480), depending upon the time of specimen testing. IgA was measured via enzyme-linked immunosorbent assay (Cincinnati Children’s Hospital Medical Center, Cincinnati, OH, USA, Lab Developed Test). Nucleocapsid testing was measured via enzyme-linked immunosorbent assay (EUROIMMUN, Mountain Lakes, NJ, USA, EI 2606-9601-2 G). Each participant was notified of SARS-COV-2 antibodies as well as present or absent nucleocapsid.

### 2.3. Statistical Analyses

**Figure 1:**
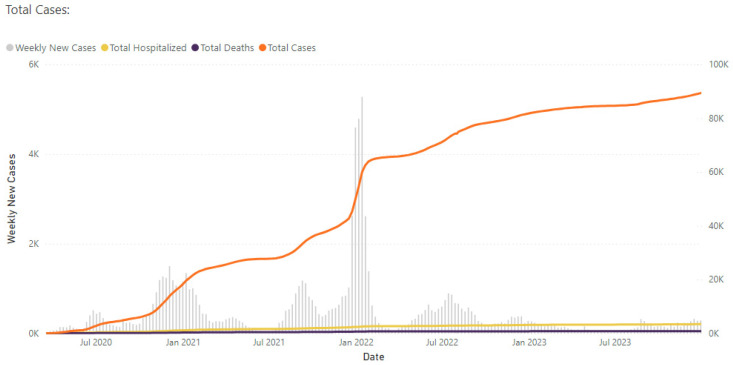
Cases, hospitalizations, and deaths from COVID-19 from July 2020 through early 2023.

Means with standard deviations and numbers with percentages were used to describe participant characteristics as well as COVID-19 symptoms and side effects for the full sample and separately for index cases and household contacts. Similarly, the percentage of participants with IgG, IgM, and IgA antibodies for COVID-19 as well as those specific to the nucleocapsid protein were described for index cases and close contacts. All testing was interpreted qualitatively as the presence of antibodies: yes/no. The presence of nucleocapsid protein among household contacts (secondary attack rate) was evaluated by demographic and sociodemographic characteristics including race, ethnicity, employment, and experience with adversity (ACEs) and discrimination. High ACEs were defined as having 4 or more, as the prior literature has described poor health outcomes in patients using this distinction [8], and this group of contacts was compared to contacts with 3 or fewer ACEs. Differences were tested for statistical significance using Fisher’s exact test, due to small sample sizes for some comparisons. To determine whether antibody status varied by test type, we summarized the presence of antibodies among index cases by PCR, antigen, or missing data for test type. In addition, to determine whether infection and antibody characteristics varied by time (or pandemic wave) due to potential differences in immune response by variant, we stratified by dates including prior to 19 June 2021 (the first study visit was 3 December 2020), 20 June 2021 to 26 March 2022, and after 26 March 2022 (the last study visit was 17 March 2023). Dates were determined by epidemiologic curves provided by the CHD (Figure 1). We calculated the secondary attack rate (based on nucleocapsid positivity) for vaccinated and unvaccinated participants and by vaccine type. Finally, to determine differences in antibody status by additional factors, we stratified analyses by COVID-19 wave, type of test (antigen versus PCR), vaccination status, and time since infection. All analyses were available case analyses, and we did not impute data.

## 3. Results

A total of 245 participants were enrolled in the study, including 146 index cases and 99 close contacts. This sample was composed of roughly 26% who identified as Black or African American and 3.4% as Hispanic adult participants (Table 1). Our sample underrepresented the Black population of Cincinnati, which is around 40%.

A majority (93.2%) of the index cases were symptomatic. The most prevalent symptoms were subjective fever, chills, muscle aches, rhinorrhea, sore throat, cough, and headache, which were each experienced by more than 50% of index cases. About one-third of the index cases reported losing their senses of taste and smell. Only one case was hospitalized, and three had an abnormal chest x-ray. Few household contacts (24.2%) experienced COVID-19 symptoms, with rhinorrhea (13.1%) and headache (17.2%) being the most prevalent (Table 2).

Most participants, including both index cases and household contacts, had IgG antibodies to COVID-19 (93.2% and 80.8%, respectively). In addition, most index cases had IgA antibodies (91.8%), while only 67.6% of household contacts had IgA antibodies. A similar proportion of index cases and household contacts had IgM antibodies (12.3% and 11.1%, respectively). The nucleocapsid results varied by case status. Among index cases, 65.8% were positive for nucleocapsid protein, while 44.4% of contacts were positive. The vaccine status was higher among cases compared with close contacts (78.8% versus 56.6%) (Table 3).

The secondary attack rate (defined here as the percentage of close contacts who contracted SARS-CoV-2 indicated by positive test for the nucleocapsid protein) was compared by race, ethnicity, sociodemographic indicators, and psychosocial variables. While 45.5% of White close contacts were positive for the nucleocapsid protein, 57.1% Black or African American contacts were positive (*p* = 0.10). The proportion was also higher for contacts who had experienced four or more ACEs compared with less than four (76.9% versus 36.1%, *p* = 0.02) and contacts who experienced everyday discrimination by race (66.7%, *p* = 0.08) (Table 4).

Several stratified analyses were performed to explore additional factors influencing differences in antibody status, such as the vaccination status, COVID-19 wave (Table 5), type of test (antigen vs. PCR, Supplemental Table S1), and time since infection. We observed that the test sensitivity increased with each subsequent wave. Specifically, we found positivity rates of 52.6%, 61.5%, and 69.3% among index cases across the three pandemic waves. For index cases, we further stratified by the type of COVID-19 test used for diagnosis. The IgG and IgA status remained high regardless of the test type (or if the test type was missing). Among cases with antigen testing, 77.8% were nucleocapsid positive, while 66.3% of PCR-confirmed cases were nucleocapsid positive (Supplemental Table S1). There were no appreciable differences by vaccination status or type (Supplemental Table S2). However, among nucleocapsid negative cases, the median days from the SARS-CoV-2 test to visit were 149 days versus 76 days among those nucleocapsid.

## 4. Discussion

In a diverse cohort of PCR- or antigen-testing confirmed cases and their close household contacts in Cincinnati, Ohio, we observed that a majority of close contacts experienced zero or few mild symptoms. However, 44% tested positive for the presence of the SARS-CoV-2 nucleocapsid protein, indicating the presence of the virus. In addition, the presence of SARS-CoV-2 was significantly higher among participants who were exposed to four or more ACEs. Though not reaching statistical significance, a similar pattern seemed to exist among Black participants and those who reported everyday racial discrimination.

The results from this study indicate that many individuals with COVID-19, as well as their household contacts, had antibodies to SARS-CoV-2 with a majority of cases being both IgG and IgA positive. There was a similarly high proportion of household contacts with IgG and IgA antibodies, though not as high as the cases. A low percentage of participants (similar between cases and contacts) were IgM positive (12.3% and 11.1%, respectively). Since the presence of these antibodies could represent an immune response to the vaccine (78.8% of cases and 56.6% of contacts received the vaccine), we additionally measured blood for the presence of the nucleocapsid protein. A positive result would be indicative of infection and not vaccination, as this protein is not a component of any of the vaccines. We found the presence of this protein in 44.4% of close contacts, a majority of whom did not experience any symptoms, representing subclinical disease. Interestingly, only 65.8% of cases were positive for the nucleocapsid protein, suggesting the test was not highly sensitive and, therefore, also suggesting that the 44.4% of asymptomatic contacts likely underrepresents the number with subclinical disease.

We conducted several sensitivity tests to understand the differences in antibody status, including stratifying analyses by COVID-19 wave, type of test (antigen versus PCR), vaccination status, and time since infection. Indeed, we identified 52.6%, 61.5%, and 69.3% positivity among index cases across the three assessed pandemic waves. This was higher among antigen test-positive cases and those with a shorter time between reported infection and their study visit. Assuming the test sensitivity is the same for cases and contacts, we do not expect any bias from test to visit differences, but we do believe nucleocapsid is underestimating the true prevalence of disease in close contacts. We found no difference by vaccination status or type.

A second study objective was to determine whether individuals who identified as being of Black race, Hispanic ethnicity, or with sociodemographic and/or psychosocial risk were more susceptible to contracting the SARS-CoV-2 virus from the index case. To examine this, we described the prevalence of the nucleocapsid protein in household contacts by race, ethnicity, smoking status, exposure to early adversity (as measured by the ACE scale), employment status, and experiences with discrimination (by quantity and type). We identified a higher proportion of individuals positive for the nucleocapsid protein who experienced high early adversity compared to those with lower adversity, and the differences were statistically significant. We also saw an indication of differences by race (57.1% of Black household contacts were nucleocapsid-positive compared with 45.5% among White contacts) and racial discrimination (66.7% among those reporting discrimination by rates 3 times per year or more frequently compared with 43.1% positivity among all close contacts); however, neither of these differences reached statistical significance. We do note that underrepresentation of Black participants may affect our findings. If Black household contacts were less likely to enroll and had higher transmission risk, our estimates of transmission for this group could be underestimated, limiting the interpretation of racial differences. As with other regions of the United States, Cincinnati experiences stark health disparities which can elevate the risk for disease transmission. Psychosocial factors, including early life adversity (ACEs) and discrimination may increase susceptibility to infection through multiple pathways. Chronic stress can lead to dysregulation of the hypothalamic–pituitary–adrenal (HPA) axis and immune modulation, including altered inflammatory responses and impaired antiviral immunity. These biological changes may increase vulnerability to infection or affect disease severity. In addition, individuals facing socioeconomic disadvantage, who also experience early life or ongoing psychosocial adversity also encounter barriers to healthcare access and reduced trust in health systems. Together, these mechanisms may affect transmission within households and communities. While our findings are preliminary, they support the importance of addressing psychosocial stressors and structural inequities as part of comprehensive public health preparedness.

Our results are similar to a case-ascertained immunosurveillance study conducted by Bhatt and colleagues in Ontaria, Canada, that identified a similar secondary attack rate (49.1%) [11]. Their study focused on a different set of risk factors to predict household transmission and identified increasing numbers of infected persons, high density households, adult-to-child transmission, and hospital admittance as risk factors for transmission [11]. Both our study and Bhatt et al.’s identified higher transmission rates than the combined effect of a 2019 metanalysis [12]. However, few studies used serology to identify subclinical cases, as our study did, along with the study by Bhatt et al. This approach was also clearly demonstrated in this large study that employed multiple methods to identify secondary cases [13].

There are several strengths to our study including the randomized recruitment of all reported cases in the City of Cincinnati and the use of serology to identify subclinical testing. That said, we do note several limitations. We experienced challenges in enrolling participants, both index cases and household contacts. While we did not have the opportunity to query reasons for non-participation, we recognize there were hesitations around leaving the house unnecessarily and, specifically, visiting a hospital where our study visit took place. We recognize that household contacts that did not participate may have had a different immunologic and symptom experience than those enrolled in the study. As a result of enrollment challenges, we had a relatively small sample across a long period of time and across multiple pandemic waves. However, this is also a strength as we observed characteristics preserved across different waves of the pandemic.

## 5. Conclusions

Our results confirm the high transmission of subclinical disease among household contacts and reinforce the importance of isolation of cases to prevent transmission regardless of vaccination status. Our results also suggest a relationship between transmission and psychosocial risk factors.

## Figures and Tables

**Table 1:** Number and proportion of demographics and household characteristics of n = 245 participants.

Characteristics	N (%)
N	245
Adults	210 (85.7)
Adult age, yr	42.8 (14.8)
Adult Race	
White	141 (67.1)
Black	55 (26.2)
Asian	5 (2.4)
American Indian	1 (0.5)
More than one race	1 (1.0)
Other/unknown	6 (2.9)
Adult Hispanic Ethnicity	7 (3.4)
Smoking Status	
Current	19 (9.0)
Past	33 (15.6)
Never	160 (75.5)
Adverse Childhood Experience (ACE)	
ACE ≥ 4	38 (18.2)
Employed at study visit	165 (81.3)
Household Size	
1	78 (54.2)
2	41 (28.5)
3	17 (11.8)
4	6 (4.2)
5	2 (1.4)
Everyday Discrimination *	
Racial	41 (16.7)
Gender	44 (18.0)
Physical appearance	19 (7.8)
Case Status	
Index	146 (59.6)
Close contact	99 (40.0)

* If participants answered that they experienced any type of discrimination 3 times/year or more frequently, they completed questions about the type of discrimination.

**Table 2:** Numbers and proportions of symptoms and side effects of n = 146 index cases and close contacts n = 99.

	Index	Contacts	Total
N	146	99	245
<10 years	23	17	40
Symptoms			
Yes	136 (93.2)	24 (24.2)	160 (65.3)
Pneumonia	4 (2.7)	0	4 (1.6)
Respiratory distress	11 (7.5)	0	11 (4.5)
Fever	59 (40.4)	1 (1.0)	60 (24.5)
Subjective fever	75 (51.4)	4 (4.0)	79 (32.2)
Chills	89 (71.0)	5 (5.1)	94 (38.4)
Chills w/shaking	37 (25.3)	2 (2.0)	39 (15.9)
Muscle ache	96 (65.8)	6 (6.1)	102 (41.6)
Runny nose	95 (65.1)	13 (13.1)	108 (44.1)
Sore throat	83 (56.9)	3 (3.0)	86 (35.1)
Cough	102 (96.2)	4 (4.0)	106 (43.3)
Shortness of breath	49 (33.6)	4 (4.0)	53 (21.6)
Nausea	32 (21.9)	1 (1.0)	33 (13.5)
Headache	100 (68.5)	17 (17.2)	117 (47.8)
Abdominal pain	23 (15.8)	6 (6.1)	29 (11.8)
Diarrhea	32 (21.9)	4 (4.0)	36 (14.7)
Loss of taste	48 (32.9)	0 (0)	48 (19.6)
Loss of smell	48 (32.9)	1 (1.0)	49 (20.0)
Fatigue	6 (4.1)	1 (1.0)	7 (2.9)
Abnormal chest X-ray	3 (2.1)	0	3
Other	17 (11.6)	1 (1.0)	18 (7.4)
Hospitalization			
Yes	1 (0.7)	0	1
ICU, intubation, ECMO	0	0	0

Abbreviations: Intensive Care Unit (ICU), Extracorporeal Membrane Oxygenation (ECMO).

**Table 3:** Number and proportion of antibody, nucleocapsid, and vaccine status of index cases and close contacts among 216 participants.

	Index	Close Contacts	Total
Antibody status			
IgG Positive	136 (93.2)	80 (80.8)	216 (88.2)
IgA Positive	134 (91.8)	67 (67.6)	201 (82.0)
IgM Positive	18 (12.3)	11 (11.1)	29 (11.8)
Nucleocapsid			
Positive	96 (65.8)	44 (44.4)	140 (57.1)
Negative	31 (21.2)	46 (46.5)	46 (46.5)
Indeterminant	19 (13.0)	9 (9.1)	28 (11.4)
Vaccine Status			
Received Vaccine	115 (78.8)	56 (56.6)	171 (69.8)
Received Pfizer	69 (60.0)	39 (69.6)	108 (63.2)
Received Moderna	37 (32.1)	14 (25.0)	51 (29.8)
Other Vaccine	8 (7.0)	2 (3.6)	10 (5.9)
Type Unknown	1 (0.9)	1 (1.8)	2 (1.2)

**Table 4:** Secondary attack rate (n (%) by race, ethnicity, and other sociodemographic variables in close contacts with nucleocapsid protein by race or other sociodemographic characteristics. Differences across groups of n = 72 close contacts identified by Fisher’s exact test.

	Nucleocapsid Status			
	Positive	Negative	Indeterminant	*p* Value
Close Contact Adults	31 (43.1)	33 (45.8)	8 (11.1)	
Adult Race				0.06
White	20 (45.5)	29 (53.7)	5 (9.3)	
Black	8 (57.1)	3 (21.4)	3 (21.4)	
Other	2 (100)			
Adult Hispanic Ethnicity	2 (40.0)	3 (9.4)	0 (0)	0.18
Smoking Status				0.59
Current	3 (50.0)	2 (33.3)	1 (16.7)	
Past	7 (58.3)	5 (41.7)	0 (0)	
Never	21 (38.2)	27 (49.1)	7 (12.7)	
Adverse Childhood Experience (ACE) n = 211				
ACE ≥ 4	10 (76.9)	2 (15.4)	1 (7.7)	0.02
ACE < 4	22 (36.1)	32 (52.5)	7 (11.5)	
Employed at Study Visit	23 (43.4)	25 (47.2)	5 (9.4)	0.56
Everyday Discrimination *				
Racial	8 (66.7)	2 (16.7)	2 (16.7)	0.05
Gender	8 (61.5)	4 (30.8)	1 (7.6)	0.37
Physical Appearance	3 (37.5)	5 (62.5)	0 (0)	0.65

* If participants answered that they experienced any type of discrimination three times/year or more frequently, they completed questions about the type of discrimination. Abbreviations: Adverse Childhood Experience (ACE).

**Table 5:** Number and proportion of antibody, nucleocapsid, and vaccine status of index cases (n = 146) and close contacts (n = 99) by COVID wave (total n = 245).

	3 December 2020–20 June 2021	20 June 2021–26 March 2022	26 March 2022–17 March 2023
Index	19 (13.0)	26 (17.8)	101 (69.2)
PCR confirmed	18 (94.7)	11 (42.3)	57 (57.4)
Antigen test confirmed	0 (0)	4 (15.4)	14 (13.9)
Missing	1 (5.3)	11 (42.3)	30 (29.7)
Contact	32 (33.3)	16 (16.2)	51 (51.5)
Antibody status—index			
IgG positive	16 (84.2)	25 (96.2)	95 (94.1)
IgA positive	15 (79.0)	24 (92.3)	95 (94.1)
IgM positive	5 (26.3)	3 (11.5)	10 (9.9)
Nucleocapsid			
Positive	10 (52.6)	16 (61.5)	70 (69.3)
Negative	7 (36.8)	5 (19.2)	19 (18.8)
Indeterminant	2 (10.5)	5 (19.2)	12 (11.9)
Vaccine status—index			
Received vaccine	8 (57.1)	22 (84.6)	91 (97.9)
Received Pfizer	6 (75.0)	14 (66.7)	49 (57.0)
Received Moderna	1 (12.5)	5 (23.8)	31 (36.1)
Other vaccine	1 (12.5)	2 (9.5)	5 (5.8)
Type unknown	0 (0)	0 (0)	1 (1.2)
Antibody status: contacts			
IgG positive	20 (62.5)	15 (93.8)	45 (88.2)
IgA positive	19 (59.4)	9 (56.3)	39 (76.5)
IgM positive	7 (21.9)	0 (0)	4 (7.8)
Nucleocapsid			
Positive	11 (34.4)	6 (37.5)	27 (52.9)
Negative	19 (59.4)	9 (56.3)	18 (35.3)
Indeterminant	2 (6.3)	1 (6.3)	6 (11.8)
Vaccine status: contacts			
Received vaccine	10 (50.0)	14 (87.5)	33 (97.1)
Received Pfizer	7 (70.0)	10 (76.9)	22 (66.7)
Received Moderna	2 (20.0)	2 (15.4)	10 (30.3)
Other vaccine	1 (10.0)	0 (0)	1 (3.0)
Type unknown	0 (0)	1 (7.7)	0 (0)

## Data Availability

Data may be made available with permission from the authors.

## Supplementary Materials

The following supporting information can be downloaded at https://www.mdpi.com/article/10.3390/epidemiologia6040091/s1, Table S1: Number and proportion of antibody status of n = 146 index cases by test type; Table S2: The secondary attack rate (based on nucleocapsid positivity) of vaccinated versus unvac-cinated contacts and comparison by vaccine type.
