# Supplementary material for: High Household Transmission Among Asymptomatic Contacts Across Pandemic Waves in Cincinnati, Ohio
Source: Epidemiologia (Basel). 2025 Dec 12;6(4):91. doi: 10.3390/epidemiologia6040091 (PMC12732174; doi:10.3390/epidemiologia6040091)
Supplement: Supplementary file 1 [file epidemiologia-06-00091-s001.zip › epidemiologia-3963616-supplementary.pdf]

Supplemental Table 1. Number and proportion of antibody status of n=146 index cases by test type.

	Index cases, N=146		
	86	18	42
Index	PCR	Antigen	Missing
IgG Positive	80 (93.0)	18 (100)	38 (90.5)
IgA Positive	77 (89.5)	18 (100)	39 (92.9)
IgM Positive	12 (14.0)	2 (11.1)	4 (9.5)
Nucleocapsid			
Positive	57 (66.3)	14 (77.8)	25 (59.5)
Negative	18 (20.9)	2 (11.1)	11 (26.2)
Indeterminant	11 (12.8)	2 (11.1)	6 (14.3)

Supplemental Table 2. The secondary attack rate (based on nucleocapsid positivity) of vaccinated versus unvaccinated contacts and comparison by vaccine type.

	Vax	Unvax		Moderna	Pfizer	Unknown/other
IgG Positive	9 (15.8)	1 (7.7)		14 (100)	39 (100.0)	3(100.0)
IgA Positive	51 (89.5)	5 (38.5)		13 (92.9)	35 (89.7)	3 (100.0)
IgM Positive	56 (98.3)	7 (53.9)		2 (14.3)	7 (18.0)	0 (0)
Nucleocapsid						
Positive	25 (43.9)	6 (46.2)		7 (50.0)	16 (41.0)	1 (33.3)
Negative	24 (42.1)	7 (53.9)		5 (35.7)	18 (46.2)	1 (33.3)
Indeterminant	8 (14.0)	0 (0)		2 (14.3)	5 (12.8)	1 (33.3)
